# *Salmonella* Control Programs in Denmark

**DOI:** 10.3201/eid0907.030024

**Published:** 2003-07

**Authors:** Henrik C. Wegener, Tine Hald, Lo Fo Wong, Mogens Madsen, Helle Korsgaard, Flemming Bager, Peter Gerner-Smidt, Kåre Mølbak

**Affiliations:** * Danish Veterinary Institute, Copenhagen, Denmark; † Statens Serum Institut, Copenhagen, Denmark

**Keywords:** Salmonella, control, swine, layer, broiler, pork, chicken, egg, serology, economy, *Synopsis*

## Abstract

We describe *Salmonella* control programs of broiler chickens, layer hens, and pigs in Denmark. Major reductions in the incidence of foodborne human salmonellosis have occurred by integrated control of farms and food processing plants. Disease control has been achieved by monitoring the herds and flocks, eliminating infected animals, and diversifying animals (animals and products are processed differently depending on *Salmonella* status) and animal food products according to the determined risk. In 2001, the Danish society saved U.S.$25.5 million by controlling *Salmonella.* The total annual *Salmonella* control costs in year 2001 were U.S.$14.1 million (U.S.$0.075/kg of pork and U.S.$0.02/kg of broiler or egg). These costs are paid almost exclusively by the industry. The control principles described are applicable to most industrialized countries with modern intensive farming systems.

Salmonellosis is one of the most common causes of foodborne diarrheal disease worldwide. Most of these infections are zoonotic and are transmitted from healthy carrier animals to humans through contaminated food. The main reservoir of zoonotic *Salmonella* is food animals, and the main sources of infections in industrialized countries are animal-derived products, notably fresh meat products and eggs. In developing countries, contaminated vegetables, water, and human-to-human transmission are believed to contribute to a comparatively larger proportion of the human cases than those in industrialized countries (*1*). However, the incidence of human salmonellosis increased in most industrialized countries in the 1980s and 1990s. Rapid spread of a limited number of successful *Salmonella* clones in different sectors of food animal production (swine, broiler chickens, and particularly layer hens) has been suggested as the most important cause of this increase (*2*).

Despite much research and many national and international attempts to implement control strategies, the incidence of human salmonellosis in most countries remains high. One notable exception is Sweden, which remains essentially free from the *Salmonella* problems typical for most other industrialized countries. The background for the Swedish success has been described (*3*). Unfortunately, other countries cannot apply the Swedish model of *Salmonella* control, which requires near freedom from *Salmonella* in domestic food animal production from the onset. In the European Union, the Zoonosis Directive (*4*) was an attempt to initiate a European Union–wide control effort against foodborne zoonoses, particularly *Salmonella* in broiler chickens and layer hens. Most European Union countries found that they either could not or would not implement the directive, which did not permit use of vaccines, antimicrobial drugs, or both as elements in the control program of *Salmonella* in broiler chickens or layer hens. This constraint was seen as an obstacle by some countries. Recently a new directive has been formulated, which is awaiting final approval by the European Union Parliament.

In Denmark, the incidence in human salmonellosis increased rapidly in the second half of the 1980s because of the spread of *Salmonella* in broiler chickens. This increase led to the initiation of a targeted national control program (*5*). Subsequent spread of *Salmonella* in swine and layer hens has also led to increases in human disease incidence and subsequently to the development and implementation of targeted control efforts (*6*–*8*). We review Denmark’s *Salmonella* control programs and the effect on *Salmonella* in food animals, food, and humans. We also evaluate and discuss control costs and public health economy aspects.

## Control of *Salmonella* in Broiler Chickens

### Objectives, Program, and Effects

The initial aim of the program was that <5% of broiler flocks would be infected with *Salmonella*. The program was successful and was gradually revised towards assurance of complete freedom from *Salmonella* in broiler production.

The program is based on the principle of top-down eradication, ensuring freedom from *Salmonella* from the top of the broiler-breeding pyramid down. Infected flocks of breeding animals are destroyed, and infected birds are processed for slaughter. The testing program has developed gradually to adjust to higher food safety objectives. As progress stalled, more intensive serologic and bacteriologic testing was developed and applied (*5*,*9*–*11*). The current testing scheme is shown in Table 1.

**Table 1:** *Salmonella* surveillance of the broiler and egg production, Denmark, 2000

Stage of production	Age or frequency	Samples taken	Method
Central rearing stations, broiler and egg sector	Day-old chickens	10 samples of crate material, 20 dead or destroyed chickens^a^	Bacteriologic
	1 wk	40 dead chickens	Bacteriologic
	2 wks	2 pairs of sock samples	Bacteriologic
	4 wks	60 fecal samples^a^	Bacteriologic
	8 wks	2 pairs of sock samples	Bacteriologic
	2 weeks before moving	60 fecal samples and 60 blood samples^ab^	Bacteriologic, serologic
Breeders (hatching egg production)-broiler and egg sector	Every 2 wks	50 dead chickens or meconium from 250 chickens taken from the hatchery^ac^	Bacteriologic
	Every wk	2 pairs of sock samples^d^	Bacteriologic
Hatchery	After each hatching	Wet dust	Bacteriologic
Rearing egg production	Day-old chickens	10 samples of crate material and 20 dead chickens	Bacteriologic
	3 wks	5x2 sock samples in floor production units or 300 fecal samples	Bacteriologic
	12 weeks	5x2 sock samples in floor production units or 300 fecal samples, and 60 blood samples^b^	Bacteriologic, serologic
Egg production	Every 9th wk for eggs sold to authorized egg-packing centers	2 pairs of sock samples in floor production units or fecal samples and egg samples	Bacteriologic, serologic
	Every 6 mo for eggs sold at barnyard sale	2 pairs of sock samples or fecal samples and egg samples	Bacteriologic, serologic

^a^Requirements of the European Union Zoonosis Directive (92/117/EEC).

^b^Samples taken by the district veterinary officer.

^c^Samples taken by the district veterinary officer every 8 weeks.

^d^Samples taken by the district veterinary officer every 3 months.

Birds from infected flocks are slaughtered on separate slaughter lines or late in the day to avoid cross-contamination. Farmers get a better price for birds from *Salmonella-*free flocks, and slaughterhouses can use the label “*Salmonella*-free” for birds that meet criteria determined by the authorities. No decontaminants, such as organic acids or chlorine, are used during carcass processing.

**Figure 1:**
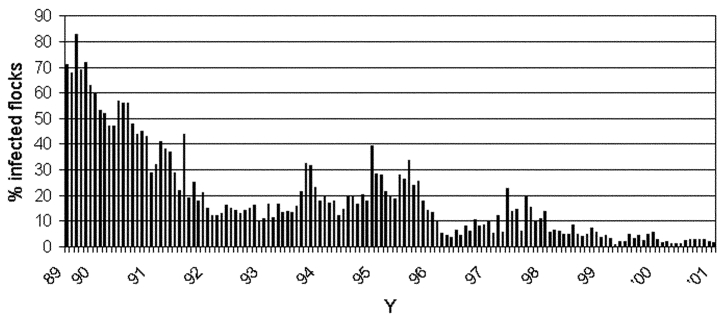
*Salmonella* in Danish broiler flocks as determined by bacteriologic testing of every flock 2–3 weeks before slaughter (N>4,000 flocks/year).

The proportion of *Salmonella*-infected broiler flocks has been markedly reduced since the initiation of the control program. Figure 1 shows that >65% of broiler flocks tested positive for *Salmonella* during the first year of the program, 1988–89, versus <5% in 2000. This decrease in *Salmonella* has led to a concomitant reduction in the proportion of infected broiler carcasses after slaughter and at retail.

The Danish government and the European Union equally compensate owners of destroyed breeding stock for their losses. In 1993, a major Danish retailer (COOP-Denmark) stopped the marketing of broiler chicken, which exceeded a 5% target. Danish chicken could not meet this target at that time, so producers suffered severe losses because they had to export their chicken to lower priced markets.

*Salmonella* can be effectively reduced (nearly eliminated) from broiler chickens by intensive flock-level testing and top-down eradication. Essential to success is a sufficiently sensitive testing program in the breeding and rearing flocks as well as in the hatcheries, i.e., one that involves intensive sampling and a combination of serologic and bacteriologic testing methods (Table 1). Bacteriologic testing alone is not sufficiently sensitive to achieve control, especially if *S.* Enteritidis infections are present. Removal of all organic material, thorough cleaning and disinfection of the poultry house, and an empty resting period of 10–14 days between flocks can effectively eliminate residual infections. In Denmark, most infections appear to be vertically transmitted (nearly always traceable to an infected hatchery or parent flock), whereas horizontal transmission from the environment and wild fauna appear to play a minor role. Competitive exclusion cultures, vaccines, or antibiotics have not been used in the Danish control program.

## Control of *Salmonella* in Layer Hens

### Objectives, Program, and Effects

**Figure 2:**
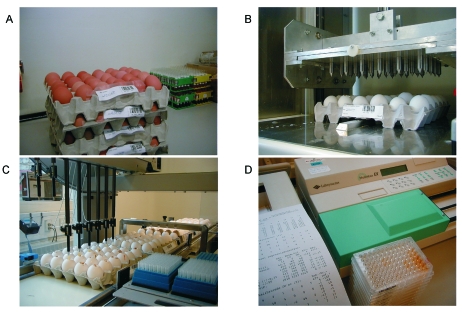
A) Receipt of 60 eggs per producer every 9 weeks (barcode indicating producer is shown). B) The “eggbreaker” punches a hole in 30 eggs at a time. C) Withdrawal of egg yolk from 30 eggs and transfer to microtiter tray. D) Enzyme-linked immunosorbent assay analysis, reading, and transfer of results to central database.

All shell eggs from commercial layer flocks should be free from *S*. Enteritidis and *S*. Typhimurium. Control of layer breeders in Denmark is essentially identical to the control program for broiler breeders (Table 1). Blood and fecal samples of rearing flocks are tested (*8*,*11*), and infected flocks are destroyed. All commercial flocks of layer hens in production are tested routinely every 9 weeks by a combination of serologic testing of egg yolk and bacteriologic testing of environmental samples (Table 1, Figure 2).

All eggs from suspect or confirmed-positive layer flocks are pasteurized. All shell eggs are distributed in a cold chain (not exceeding 12°C) and kept refrigerated at retail; eggs are generally refrigerated in private homes.

**Figure 3:**
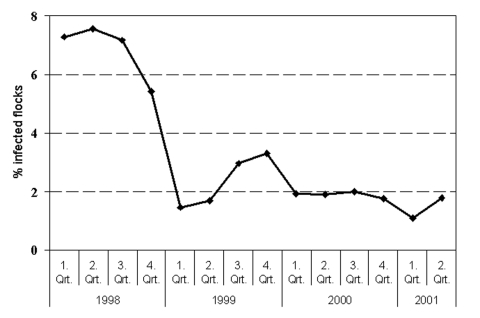
*Salmonella* in Danish layer flocks as determined by serologic and bacteriologic testing of each commercial flock in week 9 of production.

The government and the European Union equally compensate owners of destroyed breeding stock for their losses. The proportion of layer flocks infected with *Salmonella*, notably *S.* Enteritidis, has been markedly reduced since the initiation of the control program. Figure 3 shows that >7% of layer flocks tested positive for *Salmonella* in the first year of the program, 1998, versus <2% in 2001. The level of *Salmonella*-contaminated shell eggs has not been measured from the initiation of the control program. However, a year before the program began, a study of 13,000 eggs from different types of production determined the level to be 1 per 1,000 eggs (20% of the contaminated eggs harbored *S.* Enteritidis) (*12*).

Top-down eradication of *S.* Enteritidis has effectively reduced the level of *Salmonella*, notably *S.* Enteritidis, in Danish commercial layer flocks. The program has been effective in free range, deep litter, organic, and caged birds. Frequent testing by a combination of serologic and bacteriologic testing methods is essential to achieve adequate sensitivity in the monitoring program. Control of residual infections in poultry houses can be conducted with a success rate of nearly 70% by thorough cleansing and disinfection of the depopulated house (removal of all organic material, disinfection of surfaces, and resting of the empty house for 2 weeks). Day-old chicks for rearing must be antibiotic free. Competitive exclusion cultures and vaccination are not used in the Danish program. Vaccination cannot, at present, be used in combination with serologic testing because of problems of cross-reaction.

## Control of *Salmonella* in Pork

### Objectives, Program, and Effects

Denmark is the only country with a nationwide control program of *Salmonella* in pork that is integrated from “feed-to-food.” The program is based on routine testing and classification of slaughter pig herds and subsequent slaughter of pigs according to the inherent risk, as measured by the continual test program (Figure 2; Table 2). The program has been described in detail elsewhere (*7*,*8*,*13*).

**Table 2:** *Salmonella* surveillance in pig and pork production, Denmark, 2001

Type of production	Sample	No. and frequency	Response
Breeding and multiplying herds	Blood	10 times a mo	Confirmatory bacteriologic testing and restrictions on the movement of animals if above predetermined level
Pig herds	Feces	100 in 20 pools of five collected on indication	*Salmonella* reduction plan implemented
Slaughter pig producers producing >200 pigs per year	Meat juice	Depending on herd size (60–100 samples per yr). Samples are collected continuously and semi-randomly	Confirmatory bacteriologic testing (20 pools of 5 fecal samples). Herds are assigned to one of three levels depending on serology. Level 1: no sanctions; level 2: implementation of *Salmonella* reducing actions in the herd; and level 3: same as level 2 and obligatory slaughter of pigs under special hygienic precautions, including postslaughter microbial testing and potential heat treatment of all meat products
Carcass after slaughter	Surface swab	Swabs of five carcasses are pooled into one sample. One sample per day in each slaughterhouse.	Slaughterhouses exceeding a predetermined number of positive swabs in a 3-mo period are obliged to implement corrective actions

### Pre-Harvest Control

**Figure 4:**
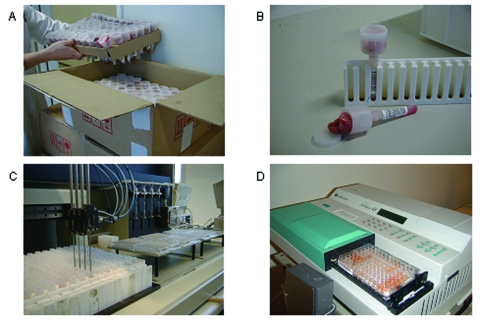
A) Receipt of pork samples from the slaughterhouse. Each tube is labeled with a barcode, indicating herd of origin. Samples are frozen overnight. B) The tube is entered in a rack with the barcode facing outward. Meat juice sieves into the tube from the container during thawing. C) Withdrawal of meat juice from tube and transfer to microtiter tray. D) Enzyme-linked immunosorbent assay analysis, reading, and transfer of results to central database.

Pigs from breeding and multiplying herds are tested monthly by serologic testing of blood samples. If a specific cutoff level is reached, bacteriologic confirmatory testing is carried out. Further, if the serologic reactions exceed a specific high level, all movement of animals is restricted. Slaughter pig herds are monitored continuously by serologic testing of “meat juice” (drip fluid released from meat after freezing and thawing) (*14*). Meat samples for testing are collected at the slaughter line, and the number of samples and frequency of sampling are determined by the size of the herd. Approximately 700,000 slaughter pigs are currently tested each year (Figure 4). Herds sending <200 pigs to slaughter each year are not tested, leaving 1.6% of the slaughter pigs outside the monitoring scheme. The herds are categorized in three levels based on the proportion of seropositive meat juice samples during the last 3 months. Owners in level 2 and 3 are encouraged to seek advice on how to reduce the *Salmonella* problem in the herd (e.g., feeding, hygiene, and management). Furthermore, payment from the slaughterhouse is reduced by 2% and 4%, respectively.

The postharvest surveillance program has been described (*8*,*15*,*16*). Pigs from herds in levels 1 and 2 are slaughtered traditionally without any special precautions. Pigs from level 3 herds can only be slaughtered in special slaughterhouses under special hygienic precautions. Carcasses from level 3 herds are tested for bacteria after slaughter, and if the level of contamination exceeds a certain limit all carcasses from the particular herd have to undergo heat treatment or other risk-reducing processing. All slaughterhouses do routine bacteriologic testing of carcasses according to a sampling plan, which ensures that testing is random and representative of the national swine production (>30,000 samples/year). Slaughterhouses that exceed a certain predetermined level of *Salmonella* in the routine monitoring of carcasses are obliged to investigate and reduce the contamination problem to an acceptable level.

**Figure 5:**
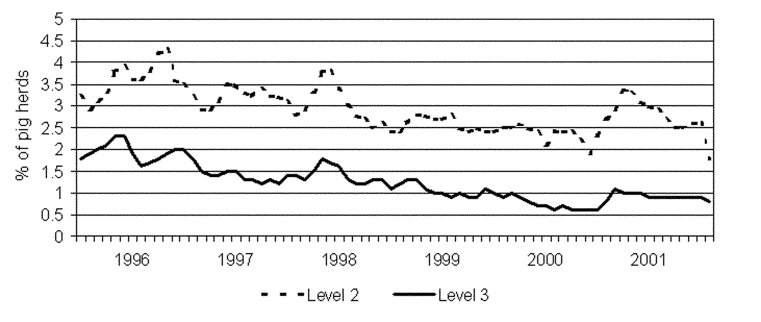
Prevalence of *Salmonella* in Danish pig herds as determined by continuous serologic testing of all commercial pig herds (N >700,000 samples tested/year). Herds are categorized in three levels based on the proportion of seropositive meat juice samples during the last 3 months. Owners in level 2 and 3 are encouraged to seek advice on how to reduce the *Salmonella* problem in the herd (e.g., feeding, hygiene, and management). Furthermore, pigs from level 3 herds can only be slaughtered in special slaughterhouses under special hygienic precautions. Data from the Danish Veterinary and Food Administration.

**Figure 6:**
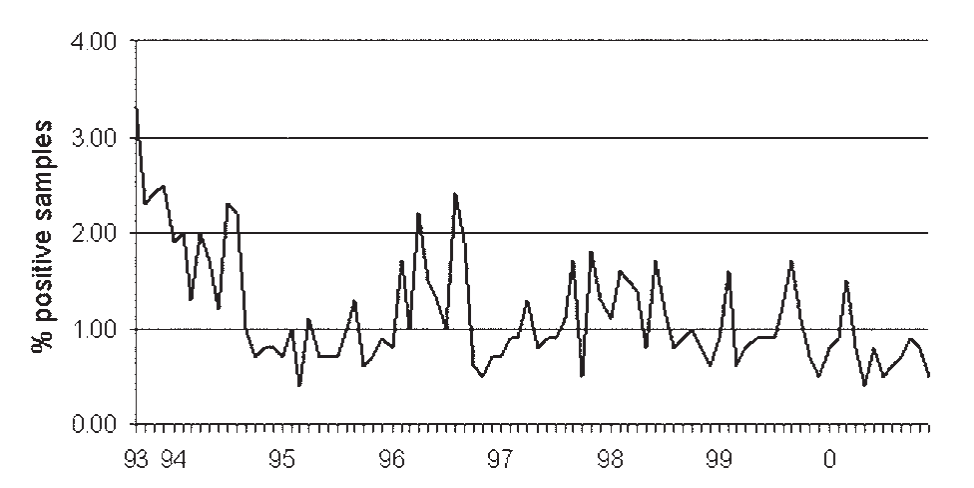
*Salmonella* detected in pork, as determined by continuous randomized sampling of pork end-products from all major national pig slaughterhouses (N >30,000 samples/year).

The prevalence of swine herds in level 2 and 3 respectively, has been steadily reduced since the program began (Figure 5). Bacteriologic testing has indicated that the herd infection level was reduced by 50% (from 14.7% to 7.2% in small herds and 22.2% to 10.4% in large herds) from 1993 (when the program was implemented) to 1998 (*17*). In the same period, the level of *Salmonella* contamination in pork products, as determined by the routine monitoring program, was reduced from 3% to <1% (Figure 6).

Because *Salmonella* eradication in swine herds is difficult because of the continual nature of the production system, reducing the infection level should be the aim of a control strategy. The low infection level in the herds and contamination in the products can likely be reduced further in Denmark. As the contamination level goes below 1%, testing for contamination requires increasingly large numbers of samples and consequently becomes very expensive, which is one reason the sampling plan of herds and products has become more sensitive in recent years. This change in testing sensitivity makes it difficult to compare current and past levels of infection and contamination but is nevertheless a necessity for the continued improvement of the program.

A combination of serologic and bacteriologic testing is essential for the success of the program. Nearly 1 million serologic samples are tested each year. Testing this large number of samples would not be possible because of financial and logistic constraints if the program were to rely on bacteriologic testing alone.

Feeding strategy (e.g., increased coarseness of feed and wet feeding) and improved management (e.g., sectioning and all-in all-out production) and hygiene standards are important elements in the preharvest control efforts. Using commercial prebiotic cultures is not necessary; natural microflora in the feed, especially wet fermented feed, appear to have a protective effect (*18*).

A reduction of *Salmonella* in slaughter pig herds has been attained without *Salmonella*–free breeding herds. However, to ensure the highest degree of consistency in the program, the levels of *Salmonella* in breeding herds should be kept as low as possible, and infected breeding herds should not be sold to producers of herds of a superior *Salmonella* status.

## Determination of Public Health Impact

To better explain the mechanisms in the occurrence of *Salmonella* infections in humans, the Danish Zoonosis Centre has previously described a method that estimates the number of human cases attributable to each of the major animal-food sources (*19*,*20*). Using this method, we compared *Salmonella* types isolated from animals and foods with *Salmonella* types isolated from humans. In brief, subtypes of *Salmonella* that are almost exclusively found in a particular food animal reservoir or food type (unique types) are used as anchor points for the distribution of subtypes occurring in several reservoirs and sources. All human infections caused by the unique types are associated with the indicated food type or derived from the indicated food animal reservoir (e.g., pork, beef, chickens, or eggs). *Salmonella* types, which occur in several reservoirs, are distributed relative to the prevalence of unique types in each reservoir or food type. Detailed knowledge of the distribution of *Salmonella* types in all relevant food animals and food types, generated through intensive and continuous monitoring, is a prerequisite for the analysis. Recently, a stochastic model based on the principles of the previous method was developed and applied. This model allows us to consider the uncertainty around the estimated parameters (*21*).

**Figure 7:**
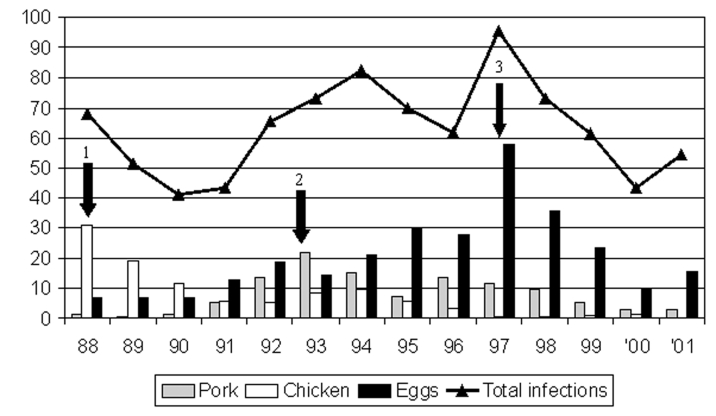
Effects of *Salmonella* control programs as indicated by incidence of human infection attributable to the different major sources of human salmonellosis in Denmark; 1) *Salmonella* control program for broiler chickens implemented, 2) *Salmonella* control program implemented for pigs and pork, 3) *Salmonella* control program implemented for layer hens and eggs The three sources account for approximately 50% to 75% of *Salmonella* each year. Remaining cases are attributable to beef, imported food products, infections acquired while traveling abroad, and unknown sources. Incidence (cases/100.000 inhabitants).

Figure 7 shows the human salmonellosis incidence associated with the three major sources of human salmonellosis in Denmark from 1988 to 2001. The year that a control program was launched for a specific food animal production system is also indicated. The control programs have been successful in achieving the main objective, a reduction of the incidence of human salmonellosis. The broiler-associated salmonellosis incidence (cases/100,000) has been reduced by >95.0%, from 30.8% in 1988 to 0.5% in 2001; the pork-associated salmonellosis incidence has been reduced by >85%, from 22.0% in 1993 to 3.0% in 2001; and the egg-associated salmonellosis incidence has been reduced by nearly 75%, from 57.7% in 1997 to 15.5% in 2001. Trends in the animal and food-specific disease incidence estimates show a high degree of agreement with the trends in prevalence of *Salmonella* in specific food animals and the corresponding animal-derived food products. These trends serve as an indirect validation of the estimates because these estimates do not rely on prevalence data.

## Economy of *Salmonella* Control

### Costs of *Salmonella* Control

The Audit Office of Denmark has evaluated the government spending in relation to the national *Salmonella* control efforts (*22*). From 1994 to 1999, the control program for broiler chickens and layer hens involved government finances of a total of 188.1 million Danish kroner (DKK) (U.S.$26.5 million) (U.S.$1.00 = 7.1 DKK). A total of 109.7 million DKK (U.S.$15.45 million) was paid to compensate farmers for destroyed animals; most of the remaining costs were associated with establishing and running the surveillance program. These costs were highest in the initial phase of the layer hen control program in 1997 but have been reduced considerably since then. In 2001, all costs associated with running the program were assumed by the poultry industry (with the exception of governments’ compensation for flocks destroyed according to the European Union Zoonosis Directive). The current control costs for layer hens and broiler chickens are estimated to be in the range of US$4.2 million per year (for 344 broiler producers, producing 135 million broiler chickens year, and 392 shell-egg producers, producing 1 billion shell-eggs per year).

In the initial phase, the *Salmonella* control program of pigs and pork cost the industry and government a total of U.S.$14 million per year. With the recent revision of the program, the responsibility has been taken over solely by the industry, and operational costs have been reduced to approximately U.S.$8.5 million per year (for 21,000 producers, producing 21–22 million slaughter pigs each year) (B. Pedersen, pers. comm.).

### Public Health Economy

Direct health costs (e.g., hospitalization, consulting a physician, and laboratory testing) as well as the costs of lost labor (e.g., loss of production per day away from work) in relation to a case of salmonellosis in Denmark were evaluated as part of a multidisciplinary task force (Korsgaard and Wegener, pers. comm.). For 2001, foodborne salmonellosis cost the Danish U.S.$15.5 million. The estimate is based on an incidence of 54.6 cases per 100,000, and approximately 10% of cases are laboratory confirmed. Assuming that 5% or 20% of cases are laboratory-confirmed changes the estimate to U.S.$25.5 million and U.S.$10.4 million, respectively.

### Costs and Benefits

Assuming that salmonellosis associated with each of the major sources would have remained at the precontrol program incidence (and not increased further) (i.e., if no action had been taken to curb the problem), we calculated a hypothetical “no-control” salmonellosis incidence. This incidence would have been 137.5 (pork 22, broiler chickens 30.8, eggs 57.7, and average residual, 27). The societal costs, in the absence of the existing control programs, would thus have been U.S.$41 million per year (assuming 10% of cases are laboratory confirmed). Thus, in 2001, Denmark saved U.S.$ 25.5 million by controlling *Salmonella.* The estimated annual *Salmonella* control costs from 2000 and onwards are approximately U.S.$14.1 million. These costs are borne almost exclusively by the animal producers and the food industry, which suggests that the costs are passed on to consumers through higher food prices. Based on the figures above and data on annual production (*23*), control costs amount to approximately U.S.$0.075/kg of pork and U.S.$0.02/kg of broiler or egg.

## Discussion

Danish *Salmonella* control efforts have been successful in achieving their objective; reduction of human salmonellosis. These efforts illustrate that with a focused and integrated programs, including a strong element of preharvest control, and based on a public-private partnership, *Salmonella* can be reduced. At the same time, the industries involved have remained profitable and internationally competitive (approximately 75% of the chicken products and 85% of the pork are exported).

Initially, the programs have received some government funding, primarily for research, development, and compensation for destroyed animals. After the initial implementation and clean-up phase of the programs, the responsibility for running and funding the programs have been nearly completely taken over by the industries involved. The government, however, maintains access to all relevant information and data through a central database managed by the Danish Zoonosis Centre, and food safety objectives continue to be determined by the Danish government.

A proactive and collaborative approach to food safety by food industry and government ensures consumers’ confidence in the domestic food production. For example, when the bovine spongiform encephalopathy (BSE) crisis hit Europe, the beef industry in most countries was adversely affected by reduced consumer demand. In Denmark, the sale of beef remained nearly unaffected by the crisis even after the first positive BSE findings occurred in Danish cattle. These steady beef sales are likely attributable to a high degree of consumer confidence in the public and private control systems.

The success of the programs supports the effectiveness of a preharvest control approach to *Salmonella*. Monitoring and intervention at the farm and in the food animal breeding systems are feasible means to achieve lasting control of the *Salmonella* problem. Development and application of a two-tiered detection system based on a combination of serologic testing and bacteriologic confirmation have been essential for the success of the programs. Serologic testing enables semi-automated mass screening of animals and eggs at a low price and with good, and in some cases superior, sensitivity. Bacteriologic testing serves to compensate for the sub-optimal specificity of a serologic-based monitoring system. The programs could not have been operated solely on the basis of bacteriologic testing because of the higher costs involved and logistical problems (i.e., screening nearly 2 million samples per year by bacteriologic testing is unrealistic). Preharvest control tools, such as vaccines, antibiotics, or competitive exclusion, are not used to control *Salmonella* in Denmark; these tools might be counterproductive, as they mask the *Salmonella* problem rather than aid in its reduction or eradication.

Evaluating the costs and benefits of the national *Salmonella* control efforts is difficult; estimating the public health and societal costs in the absence of the control program is impossible. However, this conservative estimate suggests that efforts have been cost beneficial and those benefits are likely to increase with time.
